# Promising response to a PD-1 inhibitor (sintilimab) in non-small cell lung cancer: A case report

**DOI:** 10.1097/MD.0000000000019790

**Published:** 2020-05-22

**Authors:** Lin Zhang, Wuqian Mai, Bo Hao, Wenyang Jiang, Qing Geng

**Affiliations:** a Department of Thoracic Surgery, Renmin Hospital of Wuhan University; b Department of Cardiology, Union Hospital, Tongji Medical College, Huazhong University of Science and Technology, Wuhan, China.

**Keywords:** immune checkpoint inhibitors, ICI, neoadjuvant chemotherapy, non-small cell lung cancer (NSCLC), PD-1 inhibitor, sintilimab

## Abstract

**Rationale:**

Lung cancer is the cancer with the highest incidence and mortality in China and worldwide. Among them, 85% are non-small cell lung cancer (NSCLC). No previous reports have been published to describe the clinical effect of the immune checkpoint inhibitor (ICI) sintilimab in NSCLC yet.

**Patient concerns:**

We report a case of a 64-year-old woman with a 20-day history of chest pain with computed tomography scan showing a right lower lung mass.

**Diagnoses:**

Squamous NSCLC was diagnosed by bronchoscopy.

**Interventions:**

The patient was treated with sintilimab plus nedaplatin and paclitaxel as neoadjuvant therapy for 3 cycles, followed by right thoracotomy, right middle lobectomy, right lower lobectomy, hilar lymphadenectomy, mediastinal lymphadenectomy, and pericardiostomy.

**Outcomes:**

The patient was discharged from the hospital 12 days after operation. Pathological report showed no cancer residue in the lung tissue, the bronchial stump, the anastomotic lung marginal tissue, 2nd, 4th, 7th, 9th, 10th, 11th lymph nodes or in the peribronchial lymph nodes after repeated sampling. The pathological stage was deemed T0N0M0. She remained disease free until the latest follow up in July 2019.

**Lessons:**

Sintilimab is a promising drug for patients with locally advanced NSCLC. However, its efficacy still requires further clinical investigations.

## 1 Introduction

Lung cancer has become the cancer with the highest incidence and mortality in China and worldwide, with about 1.5 million new cases in the world and around 85% of them are non-small cell lung cancer.^[1,4]^ Surgery is the first choice of treatment when cancer is resectable but only a small number of patients are suitable for it. Platinum-containing doublets are the standard plan of chemotherapy for the treatment of advanced NSCLC and preoperative chemotherapy significantly improves overall survival.^[1]^

Immune checkpoint inhibitors are drugs that disrupt inhibitory signaling or enhance stimulatory signaling in T cells. The emergence of immunotherapy has revolutionarily altered the landscape of lung cancer treatment. The most intensively studied immune checkpoint proteins, cytotoxic T-lymphocyte–associated-4 (CTLA-4) and the programmed cell death receptor-1 (PD-1), are receptors expressed on T cells that can promote cancer cell immune evasion. Among those ICI drugs approved by America Food and Drug Administration (FDA), nivolumab and pembrolizumab are PD-1 inhibitors, atezolimab is a programmed cell death-ligand 1 (PD-L1) inhibitor.^[12]^ Sintilimab, a PD-1 inhibitor approved in China for the treatment of classical Hodgkin lymphoma, is still undergoing phase I, II and III development for use in various solid tumors including NSCLC in China.^[8]^

Herein, we reported a case of locally advanced squamous lung cancer treated with neoadjuvant chemotherapy plus sintilimab and was pathologically evidenced “no tumor” with a T0N0M0 pathological stage after surgery.

## 2 Case presentation

**Figure 1:**
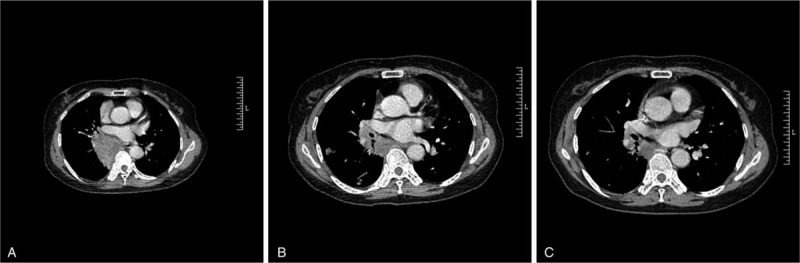
Chest CT scans on January 25, 2019 (A), March 26, 2019 (B), and April 19, 2019 (C). The tumor mass measured 6.5 cm × 5.3 cm, 2.9 cm × 2.3 cm, and 1.7 cm × 2.0 cm, respectively.

A 64-year-old woman was hospitalized in our hospital with a 20-day history of chest pain. She initially presented with episodic chest pain mainly on the right hemithorax in January 2019. Her appetite, mental status and sleep were deteriorated. There were no systemic symptoms or history of pulmonary disease. Nor were there any signs of supraclavicular lymphadenopathy. Chest computed tomography (CT) scan on January 25, 2019 showed a right lower lobe lung mass of 6.5 cm × 5.3 cm located adjacent to the pulmonary vein (Fig. 1A), with mediastinal and hilar lymphadenopathy. There was no evidence of metastasis to the abdomen, brain, or bone. Pulmonary function tests were within normal limits. Tumor marker test on January 25, 2019 revealed: CEA 51.39 ng/ml, CA125 185.4 ng/ml, NSE 27.87 ng/ml, CYFRA 6.34 ng/ml. Bronchofiberscope biopsy reported lung squamous cell carcinoma. The specimen was sent for PD-L1 antibody examination. Tumor proportion score (TPS) was 80% as reported by the result. According to the 8th edition lung cancer stage classification, her disease was staged clinically as IIIB (T3N2M0) and was therefore inoperable.^[5]^

She had no history of smoking or alcohol, nor was there a history of surgery before. Diagnose of lacunar infarction was made in 2018 by brain MRI. There was a history of hypertension for less than 1 year and nifedipine was oral administered quaque die to control her blood pressure. She had no family history of genetic disease. Her father died of pulmonary abscess and her mother died of hemoptysis (etiology unknown).

When the evaluation of surgery was made, the proximity of the tumor to the hilum increased the risk of surgery and the likelihood of right pneumonectomy. To reduce tumor size with the goal of making the tumor resectable, nedaplatin (total dose of 80 mg/m^2^ on days 2, 3, 4) and paclitaxel (total dose of 175 mg/m^2^ on day 1) were given as neoadjuvant chemotherapy in February 2019. The cycle of chemotherapy is 21 days. Meanwhile, as part of a clinical trial of Innovent Biologics company, sintilimab was given as neoadjuvant therapy every 3 weeks, with reference to the instructions (Sintilimab is recommended to be injected intravenously with a dose of 200 mg per 3 weeks). After 2 cycles of the above therapy, CT scan on March 26, 2019 showed a shrunk right lower lobe lung mass of 2.9 cm × 2.3 cm with multiple new nodules around the mass (Fig. 1B). The therapeutic effect was evaluated as PR according to the irRC. Due to the effective response, another cycle of neoadjuvant chemotherapy and sintilimab treatment was given in April 2019. After the 3rd circle of treatment, CT scan on April 19, 2019 showed a right lower lobe lung mass of 1.7 cm × 2.0 cm with multiple diminished nodules around the mass (Fig. 1C). During neoadjuvant chemotherapy and sintilimab therapy, only mild cough was reported. The evaluation of adverse effect was class I (Mild symptoms; intervention not indicated) according to the CTCAE criteria. The overall therapeutic effect was evaluated as PD according to the response evaluation criteria in solid tumors (RECIST) but was evaluated as PR according to the irRC. After the reevaluation of surgery, right thoracotomy, right middle lobectomy, right lower lobectomy, hilar lymphadenectomy, mediastinal lymphadenectomy and pericardiostomy were performed without a complication on April 26, 2019. The patient recovered steadily and was discharged home on May 8, 2019. Pathological report stated that no cancer residue was evidenced in the lung tissue after repeated sampling, no cancer was detected in the bronchial stump, anastomotic lung marginal tissue, 2nd, 4th, 7th, 9th, 10th, 11th lymph nodes or in the peribronchial lymph nodes. The tumor was pathologically staged as T0N0M0. The specimen was sent for PD-L1 examination and TPS was 0% according to the report.

At the time of follow up in July 2019, the patient underwent chest CT scan, brain CT scan, abdominal ultrasound scan, and urologic ultrasound scan. No evidence of tumor recurrence or metastasis was found in the aforementioned examinations. Tumor marker on July 23th, 2019 revealed: CEA 4.05 ng/ml, CA125 25.90 ng/ml, NSE 13.15 ng/ml, CYFRA 2.27 ng/ml. The patient remained disease free and was prescribed with careful monitoring for possible disease recurrence in every 3 months.

## 3 Discussion

Despite important advances in the treatment of NSCLC in the past decade, lung cancer remains one of the leading lethal malignancies worldwide. The neoadjuvant chemotherapy has improved survival of NSCLC patients and makes inoperable cancer operable.^[1]^ The advent of ICIs has radically changed the treatment landscape of NSCLC. Nivolumab was approved by the FDA to treat patients with metastatic squamous NSCLC in 2015.^[9]^ Prembrolizumab was approved by FDA as first-line treatment in metastatic NSCLC with high PD-L1 expression in 2016.^[12]^ The combination of pembrolizumab and chemotherapy to treat NSCLC was approved by the FDA in May 2017. The combination of atezulizumab plus bevacizumab and chemotherapy in the treatment of NSCLC was approved in October 2018.^[12]^ However, as a PD-1 antibody approved in China to treat classical Hodgkin lymphoma, sintilimab is still under clinical trials to test its efficacy and safety in solid tumors.^[3,8]^

As a part of a clinical trial of Innovent Biologics Company, sintilimab was provided to the patient and administrated with informed consent. Approval by the Committee on Ethics of Renmin Hospital of Wuhan University was obtained. After 2 cycles of neoadjuvant chemotherapy and sintilimab therapy, CT scan on March 26, 2019 showed multiple new nodules around the shrunk mass. According to the RECIST, the overall response was evaluated progressive disease (PD).^[6]^ However, this phenomenon might be a unique response pattern of immunotherapy termed pseudoprogression, which means that some patients whose disease met the criteria for disease progression based on RECIST 1.1 were noted to have late but durable responses.^[14]^ The recruitment of immune cell in the tumor tissue enhanced by ICIs caused the delay of drug effect and the emergence of new foci might be the inflammatory infiltration and local inflammatory response of previously unmeasurable foci.^[7]^ Due to the disparities between immunotherapy and traditional therapy, new criteria termed immune-related response criteria (irRC) were proposed in 2009 and its revised version in 2013.^[11,15]^ In irRC, the baseline tumor assessment was the sum of the products of the 2 largest perpendicular diameters (SPD) of all index lesions and tumor burden (TB) was defined as the sum of the SPD of index lesions and SPD of new measurable lesions. New lesions were not defined as progression but were included into tumor burden. Meanwhile, complete remission (CR), partial remission (PR) or PD should be evaluated by 2 consecutive observations not less than 4 weeks apart.^[15]^ According to the new criteria and the CT scan in April, the overall response before surgery in this case should be evaluated PR.

TPS is an evaluation marker of PD-L1 protein expression, PD-L1 positive is defined as TPS ≥ 1%, low PD-L1 expression is defined as 1% ≤ TPS < 49%, and high PD-L1 expression is defined as TPS ≥ 50%. In 2016, an open-label, phase 3 trial called KEYNOTE-024 showed that in patients with advanced NSCLC and PD-L1 expression on 50% or more of tumor cells, the humanized monoclonal antibody against PD-1 pembrolizumab was associated with significantly longer progression-free and overall survival than was platinum-based chemotherapy.^[13]^ Pembrolizumab was therefore recommended as a first line therapy in treatment-naive patients with PD-L1 TPS of 50% or greater by FDA and EMA.^[12]^ In 2019, a randomized, open-label, phase 3 clinical study KEYNOTE-042 revealed that pembrolizumab monotherapy was associated with significantly higher overall survival as compared to the platium-based chemotherapy in untreated locally advanced or metastatic non-small-cell lung cancer patients with low PD-L1 TPS (1% ≤ TPS < 49%).^[10]^ The pembrolizumab monotherapy was therefore considered to be extended as a first-line therapy for patients with locally advanced or metastatic non-small-cell lung cancer without sensitizing EGFR or ALK alterations with low PD-L1 TPS. The TPS of this patient was 80%, indicating a high PD-L1 expression and she was therefore qualified for pembrolizumab administration. However, she chose sintilimab instead of pembrolizumab because of the economic reasons and the effective therapeutic effect was proved by later gene detection and pathological report. The TPS declined to 0%, implicating the elimination of tumor cells, as evidenced by the pathological results.

Sintilimab is an anti-programmed cell death receptor-1 antibody, blocking the interaction of PD-1 with its ligands (PD-L1 and PD-L2) and consequently helping to restore the endogenous antitumor T-cell response. Although not approved by China or FDA yet, sintilimab as monotherapy or in combination with chemotherapy have displayed preliminary activity in advanced solid tumors including locally advanced squamous NSCLC in a cohort of a six-cohort, open-label, multicentre, phase Ib study (NCT02937116).^[2]^

In conclusion, this case added to an evidence of its efficacy and provided patients of locally advanced NSCLC and high expressions of PD-L1 with more abundant choices of treatment. However, more clinical trials are needed to provide stronger evidence.

## Author contributions

**Conceptualization:** Lin Zhang.

**Data curation:** Bo Hao.

**Investigation:** Wuqian Mai, Wenyang Jiang.

**Methodology:** Wuqian Mai.

**Resources:** Qing Geng.

**Supervision:** Qing Geng.

**Validation:** Bo Hao, Wenyang Jiang.

**Writing – original draft:** Lin Zhang.

**Writing – review & editing:** Qing Geng.

## References

[1] Preoperative chemotherapy for non-small-cell lung cancer: a systematic review and meta-analysis of individual participant data. Lancet 2014;383:1561–71. 24576776 10.1016/S0140-6736(13)62159-5 PMC4022989

[2] Ying K Xu N Jiang H Efficacy and safety of sintilimab combined with 1st line chemotherapy in advanced squamous cell non-small cell lung cancer [abstract no. OA08]. J Thorac Oncol 2018;13: 12 Suppl: S1047.

[3] Ansell SM Sintilimab: another effective immune checkpoint inhibitor in classical Hodgkin lymphoma. Lancet Haematol 2019;6:e2–3. 30612711 10.1016/S2352-3026(18)30210-2

[4] Chen W Zheng R Baade PD Cancer statistics in China, 2015. CA 2016;66:115–32. 26808342 10.3322/caac.21338

[5] Detterbeck FC Boffa DJ Kim AW The eighth edition lung cancer stage classification. Chest 2017;151:193–203. 27780786 10.1016/j.chest.2016.10.010

[6] Eisenhauer EA Therasse P Bogaerts J New response evaluation criteria in solid tumours: revised RECIST guideline (version 1.1). Eur J Cancer 2009;45:228–47. 19097774 10.1016/j.ejca.2008.10.026

[7] Guaitoli G Baldessari C Bertolini F Are we ready to describe response or progression to immunotherapy in lung cancer? Crit Rev Oncol/Hematol 2019;138:112–9. 10.1016/j.critrevonc.2019.04.002 31092366

[8] Hoy SM Sintilimab: first global approval. Drugs 2019;79:341–6. 30742278 10.1007/s40265-019-1066-z

[9] Kazandjian D Suzman DL Blumenthal G FDA approval summary: nivolumab for the treatment of metastatic non-small cell lung cancer with progression on or after platinum-based chemotherapy. Oncologist 2016;21:634–42. 26984449 10.1634/theoncologist.2015-0507 PMC4861371

[10] Mok TSK Wu Y Kudaba I Pembrolizumab versus chemotherapy for previously untreated, PD-L1-expressing, locally advanced or metastatic non-small-cell lung cancer (KEYNOTE-042): a randomised, open-label, controlled, phase 3 trial. Lancet 2019;393:1819–30. 30955977 10.1016/S0140-6736(18)32409-7

[11] Nishino M Giobbie-Hurder A Gargano M Developing a common language for tumor response to immunotherapy: immune-related response criteria using unidimensional measurements. Clin Cancer Res 2013;19:3936–43. 23743568 10.1158/1078-0432.CCR-13-0895 PMC3740724

[12] Proto C Ferrara R Signorelli D Choosing wisely first line immunotherapy in non-small cell lung cancer (NSCLC): what to add and what to leave out. Cancer Treat Rev 2019;75:39–51. 30954906 10.1016/j.ctrv.2019.03.004

[13] Reck M Rodríguez-Abreu D Robinson AG Pembrolizumab versus chemotherapy for PD-L1–positive non–small-cell lung cancer. N Engl J Med 2016;375:1823–33. 27718847 10.1056/NEJMoa1606774

[14] Seymour L Bogaerts J Perrone A iRECIST: guidelines for response criteria for use in trials testing immunotherapeutics. Lancet Oncol 2017;18:e143–52. 28271869 10.1016/S1470-2045(17)30074-8 PMC5648544

[15] Wolchok JD Hoos A O’Day S Guidelines for the evaluation of immune therapy activity in solid tumors: immune-related response criteria. Clin Cancer Res 2009;15:7412–20. 19934295 10.1158/1078-0432.CCR-09-1624

